# Persistent Intestinal Colonisation and Systemic Immune Activation Following Experimental *Campylobacter jejuni* Infection in Broiler Chickens

**DOI:** 10.3390/foods15142518

**Published:** 2026-07-16

**Authors:** Chiara Di Pancrazio, Mirella Luciani, Maria Schirone, Francesca Marotta, Carmine Merola, Antonio Cocco, Vincenzo D’Innocenzo, Elisa Di Domenico, Roberta Di Romualdo, Antonio Petrini, Flavio Sacchini, Cecilia Villani, Fabrizia Perletta, Marta Maggetti, Cristina Marfoglia, Ivanka Krasteva, Eugenio Felicioni, Stefania Salucci, Antonello Paparella, Giuliano Garofolo

**Affiliations:** 1 Istituto Zooprofilattico Sperimentale dell’Abruzzo e del Molise “G. Caporale”, Via Campo Boario, 64100 Teramo, Italy; c.dipancrazio@izs.it (C.D.P.); m.luciani@izs.it (M.L.); cmerola@unite.it (C.M.); a.cocco@izs.it (A.C.); vdinnocenzo@gmail.com (V.D.); a.petrini@izs.it (A.P.); f.sacchini@izs.it (F.S.); f.perletta@izs.it (F.P.); m.maggetti@izs.it (M.M.); c.marfoglia@izs.it (C.M.); i.krasteva@izs.it (I.K.); s.salucci@izs.it (S.S.); 2 Department of Bioscience and Technology for Food, Agriculture and Environment, University of Teramo, Via R. Balzarini, 64100 Teramo, Italy; apaparella@unite.it; 3 National Reference Laboratory for Campylobacter, Istituto Zooprofilattico Sperimentale dell’Abruzzo e del Molise “G. Caporale”, Via Campo Boario, 64100 Teramo, Italy; f.marotta@izs.it (F.M.); e.didomenico@izs.it (E.D.D.); r.diromualdo@izs.it (R.D.R.); c.villani@izs.it (C.V.); e.felicioni@izs.it (E.F.); g.garofolo@izs.it (G.G.); 4 Department of Veterinary Medicine, University of Teramo, 64100 Teramo, Italy

**Keywords:** *Campylobacter jejuni*, foodborne zoonoses, poultry, systemic immunity, cytokine profiling, chemokine response, IgY

## Abstract

Campylobacteriosis is one of the leading foodborne bacterial illnesses worldwide, with poultry meat representing its principal source of human infection. Broiler chickens are a key reservoir for *Campylobacter jejuni*, where the bacterium persists in the gastrointestinal tract without disease. Understanding this host–pathogen interaction is essential to clarify mechanisms of persistence and food safety implications. This study investigated persistent *C. jejuni* colonisation and systemic immune responses in experimentally infected broiler chickens. Following oral challenge with the virulent ST-403 strain, animals were monitored under controlled conditions. Caecal bacterial loads were determined at 13, 27, 35 and 41 days post-infection, alongside serum total and antigen-specific IgY levels and circulating cytokines and chemokines using ELISA and multiplex assays. *C. jejuni* rapidly colonised the caecum and persisted throughout the study, although bacterial loads gradually declined over time but remained high. Total IgY increased early after infection and then stabilized. Antigen-specific antibodies were higher in infected chickens than in controls. Cytokine profiling revealed distinct temporal patterns, with increased IL-6 and MIP-1β, decreased IL-2 and MIP-3α, and persistently low IFN-γ levels. *C. jejuni* established stable intestinal colonisation with a detectable but non-clearing systemic immune response. These findings further support the role of broiler chickens as a persistent reservoir for human exposure to *C*. *jejuni*.

## 1. Introduction

Foodborne pathogens represent a major global public health concern, causing millions of illnesses and thousands of deaths annually. According to the latest estimates released by the World Health Organization (WHO), approximately one in nine people worldwide becomes ill each year following the consumption of contaminated food, resulting in an estimated 866 million cases of foodborne disease and 1.52 million deaths annually [1,2]. Consequently, ensuring food safety remains a critical priority for consumers, the food industry, and the public health authorities worldwide. Identifying the principal animal and food reservoirs of zoonotic pathogens and implementing effective preventive strategies are essential to reduce the burden of foodborne diseases.

Among zoonoses, campylobacteriosis is currently the most frequently reported foodborne gastrointestinal infection in the European Union (EU). According to the 2024 report of the European Food Safety Authority (EFSA) and the European Centre for Disease Control and Prevention (ECDC), 168,396 confirmed human cases were reported, corresponding to a notification rate of 55.3 cases per 100,000 population. This value represents an 11.9% increase compared with the 2023 notification rate (49.4 per 100,000 population), confirming a statistically significant upward trend observed during the 2020–2024 period. In 2024, *Campylobacter* spp. was detected in 0.36% of 2517 ready-to-eat (RTE) food sampling units. Non-RTE food (*N* = 23,195 sampling units) had a higher contamination rate (18.5%), particularly in meat and meat products, followed by fish and fishery products–non-RTE (10.1%). *Campylobacter* spp. was detected across all fresh meat types, with the highest contamination rates observed in broiler and turkey meat, at 22.8% and 26.0%, respectively [3].

*Campylobacter* spp. is widespread among commercial poultry species, with broiler chickens representing the main reservoir associated with human infection due to the high global consumption of poultry meat [4]. Poultry consumption has been estimated to account for 18–72% of human campylobacteriosis cases. Furthermore, global broiler meat consumption is projected to increase by 17.8% by 2030 [5], potentially contributing to a further rise in the incidence of campylobacteriosis.

In broiler chickens, *Campylobacter* spp., particularly *Campylobacter jejuni*, behaves as a commensal microorganism capable of reaching concentrations of up to 10^10^ CFU/g of intestinal contents [6,7,8]. The bacterium primarily colonizes the caeca and cloaca, adhering to the mucus layer without significantly invading the intestinal epithelium [9]. Unlike its pathogenic behaviour in humans, *C. jejuni* colonisation in chickens is generally not associated with clinical signs or significant intestinal lesions. Histopathological investigations have shown minimal or no alterations in intestinal crypt architecture or tissue integrity in infected chickens [10,11]. Following introduction into a flock, the bacterium spreads rapidly, colonising most animals within a few days [12]. Nevertheless, colonisation levels may vary among individuals, and not all chickens within an infected flock are uniformly or consistently colonised [13]. Maternal antibodies are believed to partially inhibit early *C. jejuni* colonisation, which generally becomes detectable after 2–3 weeks of age [14,15]. Although bacterial shedding may decline as birds age, persistent intestinal colonisation is commonly observed. This long-term host–pathogen equilibrium suggests that the avian immune response may be insufficient to completely eliminate the bacterium, thereby allowing *Campylobacter* persistence without inducing evident pathological alterations in the host [16,17]. As a result, broiler chickens constitute an important reservoir of *Campylobacter* spp., with significant implications for food safety and public health.

Despite the extensive characterisation of intestinal colonisation dynamics, the systemic immune mechanisms underlying the persistent interaction between *C*. *jejuni* and broiler chickens remain incompletely understood. Although previous studies have investigated bacterial persistence or individual aspects of the immune response, comparatively few have integrated longitudinal assessment of intestinal colonisation with systemic humoral and cytokine profiling under controlled experimental conditions. Experimental and field studies indicate that infection induces measurable immune activation [18,19,20]; however, this response appears insufficient to achieve bacterial clearance, thereby promoting prolonged bacterial carriage within the avian gastrointestinal tract.

A better understanding of the balance between immune activation and bacterial tolerance is essential to clarify the mechanisms that allow *C. jejuni* persistence despite the development of humoral and cellular immune responses. In particular, the characterisation of systemic immune mediators, including total IgY levels, pathogen-specific antibodies, cytokines and chemokines, may provide valuable insights into the regulation of this host–pathogen interaction.

Therefore, the aim of the present study was to investigate the systemic humoral and cytokine responses in broiler chickens experimentally infected with *C. jejuni* strain 8264ST-403 complex, a virulent lineage capable of direct transmission from livestock reservoirs to humans [21], in order to better characterise the immunological mechanisms associated with bacterial persistence and their potential implications for food safety.

## 2. Materials and Methods

### 2.1. Infection, Animal Management and Sampling

All animal procedures were performed in accordance with European Directive 2010/63/EU and the Italian Legislative Decree No. 26/2014 governing the protection of animals used for scientific purposes. The experimental protocol was approved by the Italian Ministry of Health under authorization No. 445/2023-PR.

A total of 108 one-day-old unvaccinated Ross 308 broiler chicks, obtained from a commercial hatchery, were housed in two separate floor pens containing clean pine shavings under environmentally controlled conditions appropriate for their age. Upon arrival, chick tray papers tested negative for *Campylobacter* spp.

Birds were randomly allocated into two groups: a control group (20 chickens, not inoculated) and an infected group (88 chickens, inoculated). Chickens received a conventional commercial corn–soybean diet formulated to meet industry nutritional recommendations. The diet included corn, dehulled and toasted genetically modified soybean meal, wheat, toasted genetically modified soybeans, maize gluten meal, animal fats, sunflower meal, peas, hydrolyzed pork protein, dicalcium phosphate, calcium carbonate, sodium chloride, sodium bicarbonate, vitamins, trace elements, digestibility enhancers, and coccidiostats. Feed and water were provided ad libitum throughout the experimental period. Environmental conditions, including ambient temperature, ventilation, and photoperiod, were maintained according to standard broiler husbandry practices throughout the experiment.

Following a 24 h acclimation period, chickens assigned to the infected group were orally inoculated with 0.1 mL of a suspension containing 10^6^ CFU/mL of *C. jejuni* strain 8264ST-403 complex. To confirm successful intestinal colonisation, five infected chickens were euthanized at 5 dpi (days post-infection). In infected chickens, *C. jejuni* counts reached 6.72 ± 0.74 log_10_ CFU/g of caecal content, whereas *Campylobacter* spp. was not detected in the control group.

Caecal samples were collected from 20 infected broilers euthanized at 13, 27, and 35 dpi, and from 28 infected broilers euthanized at 41 dpi. Mean body weights at the sampling time points were 520, 1260, 1830, and 3570 g, respectively. In the control group, necropsies were performed on 10 birds per time point at 35 and 41 dpi, resulting in a total of 20 animals. Serum samples collected from all 20 control birds were used as the negative reference group for comparisons with infected chickens at each sampling time point, because non-inoculated birds were expected to remain free of *C. jejuni* colonization and therefore not to develop a pathogen-specific humoral response.

### 2.2. Serum Collection and Preparation

Blood samples were collected by cardiac puncture immediately after euthanasia at each sampling time point. Samples were allowed to clot at room temperature for 2 h and were then centrifuged at 1000× *g* for 15 min at 4 °C. Serum was separated, aliquoted into 2 mL tubes, and stored at −80 °C until analysis. A total of 88 serum samples were collected from infected broilers (20 birds euthanized at 13, 27, and 35 dpi, and 28 birds euthanized at 41 dpi), together with 20 serum samples obtained from uninfected control broilers.

### 2.3. Detection and Enumeration of Campylobacter spp.

Detection and enumeration of *Campylobacter* spp. from caecal contents were performed according to ISO 10272-1:2017/Amd1:2023 and ISO 10272-2:2017/Amd1:2023 [22,23].

For qualitative detection, caecal contents were directly streaked onto modified Charcoal Cefoperazone Deoxycholate agar (mCCD) and incubated at 41.5 ± 1 °C for 44 ± 4 h under microaerophilic conditions. Presumptive *Campylobacter* colonies were confirmed according to ISO procedures.

For quantitative enumeration, one g of caecal content was aseptically homogenized in 9 mL of peptone-salt solution to obtain a 10^−1^ dilution. Serial decimal dilutions were prepared, and 0.1 mL aliquots of each dilution were surface-plated in duplicate onto mCCD agar. Plates were incubated at 41.5 ± 1 °C for 44 ± 4 h under microaerophilic conditions. Following incubation, colony-forming units (CFU/g) of caecal content were enumerated, and results were expressed as mean ± standard deviations (SD). Five presumptive colonies from each plate were confirmed as *Campylobacter* spp. according to ISO criteria.

### 2.4. Species Identification by MALDI-TOF MS

Species identification was performed by matrix-assisted laser desorption/ionization time-of-flight mass spectrometry (MALDI-TOF MS). A single colony cultured on mCCD agar was directly transferred onto a MALDI target plate without prior protein extraction. Each spot was overlaid with 1 μL of matrix solution containing α-cyano-4-hydroxycinnamic acid and allowed to air-dry at room temperature before analysis.

Mass spectra were acquired using a MALDI-TOF mass spectrometer (Bruker Daltonics GmbH, Bremen, Germany) and compared with reference spectra contained in the Bruker reference database using MALDI Biotyper MBT Compass 4.1 and MBT Explorer software 4.1 (Bruker Daltonics, Bremen, Germany). Species-level identification was considered reliable when a score value ≥ 2.0 was obtained.

### 2.5. Quantification of Total Serum IgY by ELISA

Total serum IgY concentrations were quantified in serum samples collected from chickens at 13, 27, 35, and 41 dpi using a Chicken IgY ELISA Kit (Abcam, Cambridge Biomedical Campus, Cambridge, UK), according to the manufacturer’s instructions. All standards and diluted serum plates were analysed in duplicate. Absorbance was measured at 450 nm using a TECAN Sunrise microplate reader (Tecan Group Ltd., Männedorf, Switzerland), and IgY concentrations were calculated from the standard curve using a four-parameter logistic regression analysis.

### 2.6. Quantification of Anti-C. jejuni IgY Antibodies by ELISA

#### 2.6.1. Quantification of *C. jejuni* Protein Extract by BCA Assay

*C. jejuni* 8264ST-403 complex was cultured on Columbia Blood Agar (Liofilchem, Roseto degli Abruzzi, Italy) for 24 h at 41.5 ± 1 °C under microaerophilic conditions and subsequently suspended in phosphate-buffered saline (PBS). Total bacterial proteins were obtained by sonication using the Bioruptor^®^ XL system (Europe Diagenode SA, Liege, Belgium). Briefly, bacterial cells were collected by centrifugation at 1000× *g* for 10 min at 4 °C, washed twice with PBS, and resuspended in PBS until an optical density at 600 nm (OD_600nm_) of 3.0 was reached. Samples were then sonicated at high power for 15 min, and cellular debris was removed by centrifugation at 15,000× *g* for 15 min at 4 °C. Protein concentrations were quantified using the Pierce^TM^ BCA Protein Assay kit (Thermo Fisher Scientific, Waltham, MA, USA) according to the manufacturer’s instructions. Protein extracts were aliquoted and stored at −80 °C until use.

#### 2.6.2. Determination of Anti-*C. jejuni* IgY Antibodies by Indirect ELISA

Polysorp microplates (Thermo Fisher Scientific, Waltham, MA, USA) were coated overnight at 4 °C with 100 µL per well of *C. jejuni* protein extract (10 µg/mL) diluted in carbonate–bicarbonate buffer. Plates were washed five times with PBS containing 0.05% Tween-20 (PBS-T) and blocked with PBS supplemented with 2% bovine serum albumin (BSA) for 30 min at room temperature.

Following washing, serum samples diluted 1:100 in PBS-T containing 2% BSA were added (100 µL/well) and incubated for 1 h at room temperature. Plates were then washed five times and incubated for 1 h with horseradish peroxidase (HRP)-conjugated rabbit anti-chicken IgY secondary antibody (Abcam, Cambridge, UK) diluted 1:5000 in PBS-T containing 2% BSA. After the final washing steps, tetramethylbenzidine (TMB) substrate solution was added and incubated for 15 min in the dark. The reaction was stopped using 2 N sulfuric acid, and absorbance was measured at 450 nm using a TECAN Sunrise microplate reader (Tecan), as previously described [24].

### 2.7. Quantification of Cytokines and Chemokines in Chicken Serum

To investigate the systemic immune responses associated with *C. jejuni* infection, a panel of cytokines and chemokines involved in both pro-inflammatory and regulatory immune pathways was quantified in serum samples collected from infected and control chickens at 13, 27, 35, and 41 dpi. Concentrations of IL-6, IFN-α, IFN-γ, IL-10, IL-21, IL-2, M-CSF, MIP-3α, MIP-1β, RANTES, and VEGF were determined using Luminex xMAP technology with the MILLIPLEX^®^ Chicken Cytokine/Chemokine Panel kit (Invitrogen, Thermo Fisher Scientific, Vienna, Austria), according to the manufacturer’s instructions. Cytokine and chemokine concentrations were expressed as pg/mL.

### 2.8. Statistical Analysis

Data distribution was assessed for normality and lognormality using the D’Agostino–Pearson, Anderson–Darling, Shapiro–Wilk, and Kolmogorov–Smirnov tests, as appropriate. Data showing a lognormal distribution were analysed using Welch’s ANOVA with the Brown–Forsythe correction, followed by Dunnett’s T3 multiple comparisons test. Variables that did not satisfy the assumptions for parametric analysis were analysed using the non-parametric Kruskal–Wallis test followed by Dunn’s multiple comparisons test with adjusted *p*-values. Cytokines and chemokines were analysed individually rather than as a combined dataset; therefore, multiple-comparison adjustment was applied within each analyte using Dunn’s multiple comparisons test. Anti-*C. jejuni* IgY antibody levels were compared between control and infected groups at each sampling time point using Welch’s *t*-test. All statistical analyses were conducted using GraphPad Prism version 10.6.1 (GraphPad Software, San Diego, CA, USA). A two-tailed *p* value < 0.05 was considered statistically significant.

## 3. Results

### 3.1. Detection and Enumeration of C. jejuni in Caecal Content

All caecal contents and boot swab samples collected at each sampling time were positive for *C. jejuni* as confirmed by MALDI-TOF identification.

**Figure 1:**
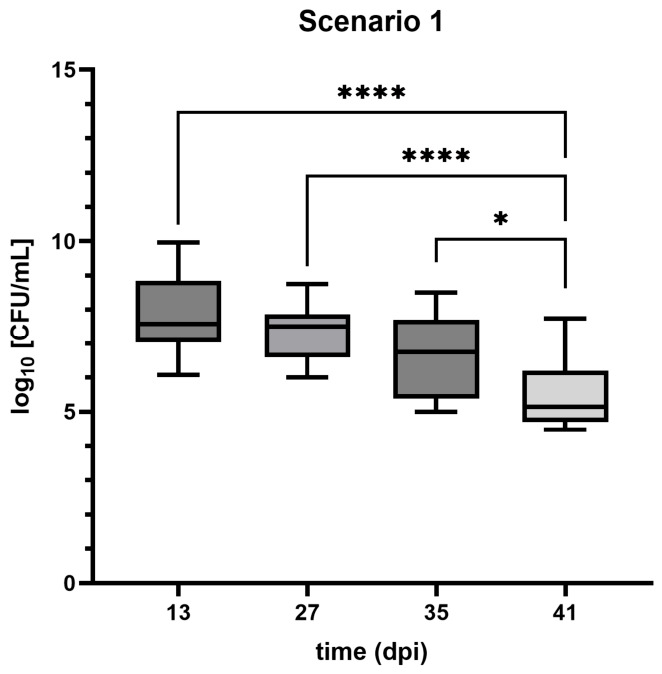
*C. jejuni* loads in the caecal contents of infected broiler chickens at 13, 27 and 35 dpi (20 chickens per time point), and at 41 dpi (28 chickens). Boxes represent the interquartile range, the central line indicates the median, and whiskers represent the full range. Statistical analysis was performed using the Kruskal–Wallis test followed by Dunn’s multiple comparisons test (adjusted *p*-values). A non-parametric approach was selected because bacterial count data did not satisfy the assumptions required for parametric analysis. * *p* = 0.0103; **** *p* < 0.0001.

Quantification of *C. jejuni* in caecal contents at 13, 27, 35, and 41 dpi is shown in Figure 1. Bacterial loads differed significantly among sampling times (Kruskal–Wallis test, *p* < 0.05). Post hoc analysis (Dunn’s test with Bonferroni correction) showed that bacterial counts at 41 dpi were significantly lower than those at 13 dpi (*p* < 0.0001), 27 dpi (*p* < 0.0001), and 35 dpi (*p* < 0.05). No significant differences were observed among 13, 27, and 35 dpi. Median bacterial loads decreased progressively over time, with the highest variability observed at 35 dpi.

### 3.2. Total Serum IgY Kinetics

**Figure 2:**
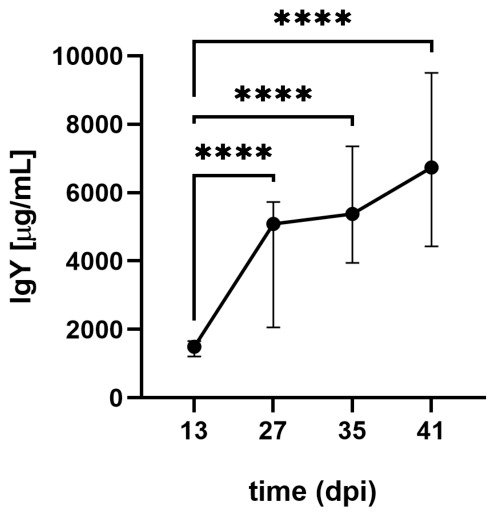
Total serum IgY concentrations in infected broiler chickens sampled at 13, 27, 35, and 41 dpi. Data are shown as geometric mean ± 95% confidence interval (CI). Because IgY concentrations followed a lognormal distribution, data were analysed using one-way Welch’s ANOVA with the Brown–Forsythe correction, followed by Dunnett’s T3 multiple comparisons test. **** *p* < 0.0001 vs 13 dpi.

Total serum IgY concentrations were measured in infected broiler chickens at 13, 27, 35, and 41 dpi. Data distribution was assessed prior to analysis and IgY concentrations were found to follow a lognormal distribution. Accordingly, data were analysed using one-way Welch’s ANOVA with the Brown–Forsythe correction, followed by Dunnett’s T3 multiple comparisons test. Total serum IgY concentrations increased significantly from 27 dpi onward compared with 13 dpi (*p* < 0.0001). No significant differences were observed among 27, 35, and 41 dpi, indicating that IgY levels remained stable after the initial increase (Figure 2).

### 3.3. Humoral Response Against C. jejuni

**Figure 3:**
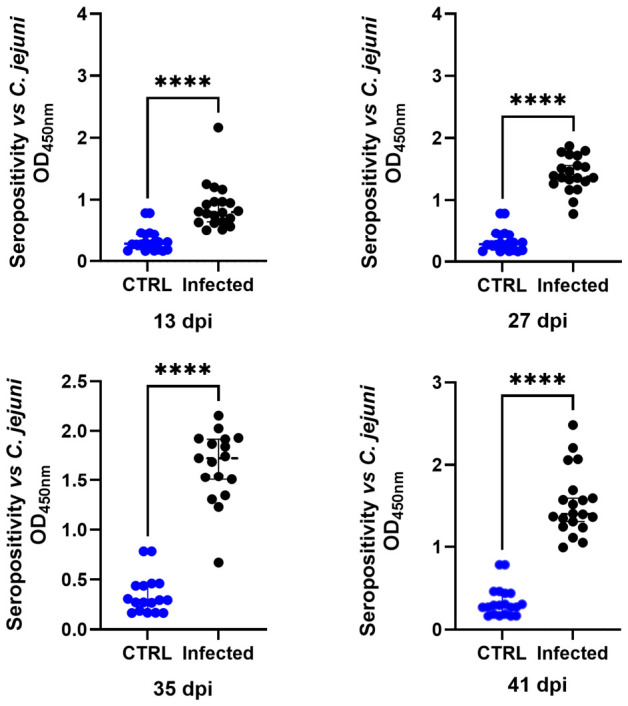
Serum IgY reactivity against *C. jejuni* antigens in infected broiler chickens sampled at 13, 27, 35 dpi (20 chickens per time point) and at 41 dpi (28 chickens), compared with non-infected (CTRL) chickens. The control group consisted of 20 non-inoculated chickens sampled at 35 and 41 dpi (*n* = 10 per time point). Serum samples from all control birds were used for comparisons with infected chickens at each sampling time point. Statistical comparisons between infected and control groups were performed using Welch’s *t*-test. **** *p* < 0.0001.

Serum reactivity against *C. jejuni* protein extracts was evaluated by indirect ELISA. Infected chickens showed significantly higher OD_450nm_ values compared with control chickens (CTRL) at all time points (Welch’s *t*-test, *p* < 0.0001), indicating the induction of a specific humoral response following infection (Figure 3).

### 3.4. Serum Cytokine and Chemokine Profile

**Figure 4:**
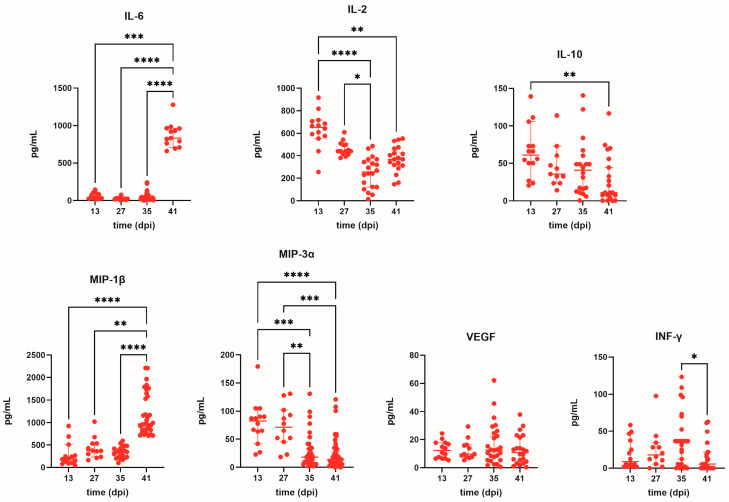
Circulating cytokine and chemokine profiles in infected broiler chickens sampled at 13, 27, 35, and 41 dpi. Data are shown as median and interquartile range. Different *Y*-axis scales were used across panels to accommodate the different concentration ranges of individual analytes. Because the data did not satisfy the assumptions for parametric analysis, statistical comparisons were performed using the Kruskal–Wallis test followed by Dunn’s multiple comparisons test with adjusted *p*-values. * *p* < 0.05; ** *p* < 0.01; *** *p* < 0.001; **** *p* < 0.0001.

Serum cytokine and chemokine concentrations were measured in infected chickens at 13, 27, 35, and 41 dpi, and compared with non-infected controls. Cytokines and chemokines were analysed individually. Dunn’s multiple comparisons test was used to adjust for multiple pairwise comparisons within each analyte. No additional correction across different cytokines or chemokines was applied because each analyte was considered an independent biological endpoint and the analyses were interpreted as exploratory. Within infected chickens, IL-6 and MIP-1β increased progressively over time, reaching higher levels at later time points. In contrast, IL-2 and MIP-3α showed a decreasing trend from early to late infection. IL-10 displayed moderate temporal variation, whereas VEGF exhibited limited and inconsistent changes. IFN-γ remained low throughout experimental period, with minimal overall variation. A significant difference was observed only between 35 and 41 dpi (Figure 4).

**Figure 5:**
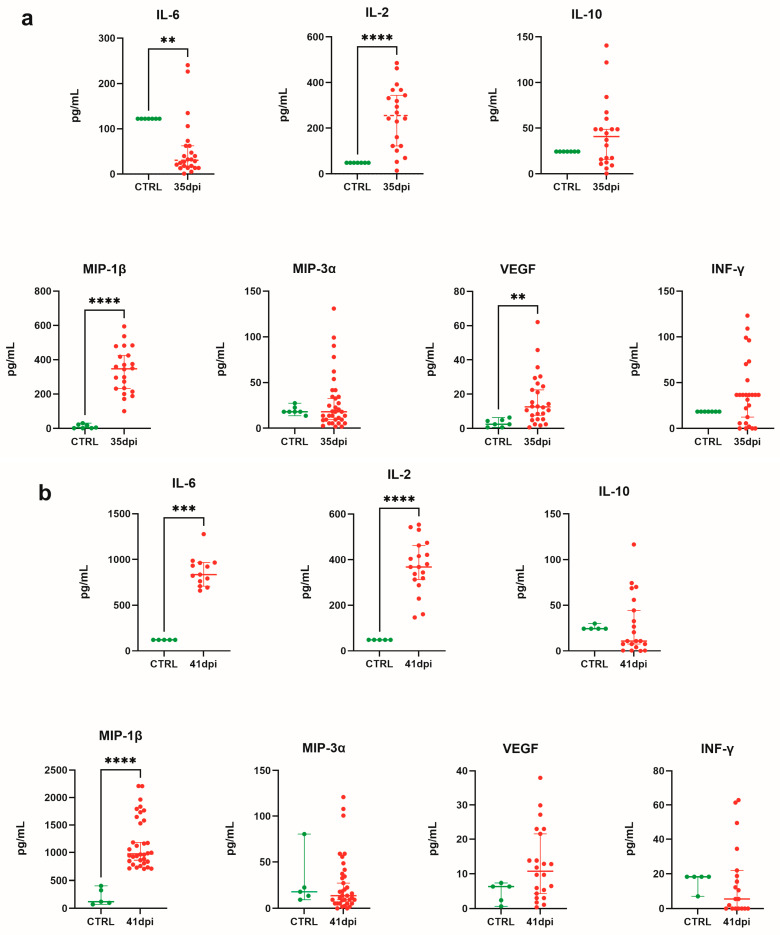
Circulating cytokine and chemokine concentrations in control and infected broiler chickens sampled at 35 dpi (**a**) and 41 dpi (**b**). Data are reported as median and interquartile range. Different *Y*-axis scales were used across panels to accommodate the different concentration ranges of individual analytes. Because the data did not satisfy the assumptions for parametric analysis, comparisons between groups were performed using the two-tailed Mann–Whitney test. ** *p* < 0.01; *** *p* < 0.001; **** *p* < 0.0001.

Comparison between infected and control groups at 35 and 41 dpi (Figure 5a,b) showed higher concentrations of IL-6, IL-2, and MIP-1β in infected chickens. VEGF showed a moderate increase at selected time points, whereas IL-10, MIP-3α, and IFN-γ showed high variability and no consistent difference between groups.

Overall, infection was associated with a detectable but heterogeneous modulation of circulating cytokine and chemokine profiles over time.

## 4. Discussion

Experimental infection with *C. jejuni* strain 8264ST-403 complex induced a measurable systemic immune response in broiler chickens, reflecting the host–pathogen interaction occurring in parallel with persistent intestinal colonisation and sustained bacterial carriage.

Total serum IgY levels significantly increased after infection, with a marked rise between 13 and 27 dpi, followed by persistently elevated levels up to 41 dpi. This kinetic profile suggests rapid activation of the adaptive humoral response and sustained antibody production during persistent intestinal colonisation. Consistently, infected birds exhibited significantly higher serum reactivity against *C. jejuni* antigens compared with controls, confirming induction of a pathogen-specific antibody response. These findings suggest that systemic antibody responses primarily reflect immune recognition of the pathogen rather than an effective mechanism leading to bacterial clearance in the avian host. This pattern is consistent with persistent intestinal colonisation and ongoing bacterial shedding, with potential implications for contamination during slaughter and processing. However, despite the clear humoral activation, colonisation was not eliminated, indicating that antibody-mediated responses alone may be insufficient to clear bacteria in broiler chickens, although the present study did not directly evaluate the functional protective activity of these antibodies [25].

At the cytokine level, infection was associated with a moderate and temporally regulated modulation of circulating immune mediators. Cytokine profiling was specifically focused on the later time points (35 and 41 dpi) to characterise the immune response during established colonisation, when host–pathogen interactions have reached a more stable equilibrium [20]. IL-6 and IL-2 were increased in infected birds, suggesting systemic immune activation and involvement of T-cell-associated pathways.

The upregulation of chemokines such as MIP-1β and MIP-3α indicates enhanced leukocyte recruitment and inflammatory signalling, while the increase in VEGF at later time points may reflect vascular adaptation or tissue remodelling associated with sustained immune stimulation. IL-10 modulation also indicates that immune activation was accompanied by regulatory mechanisms, suggesting a balanced rather than excessive inflammatory response, which may allow bacterial persistence while limiting host pathology and sustaining a carrier state relevant for downstream contamination risk in poultry productions systems [26]. Although several cytokines exhibited statistically significant temporal changes, the magnitude of these differences remained relatively modest, consistent with a detectable but limited systemic inflammatory response associated with persistent colonisation.

In contrast, IFN-γ showed only limited modulation, with low circulating levels throughout the experimental period and minor temporal variation. This finding is compatible with a limited systemic Th1-associated response during persistent *C*. *jejuni* colonisation. However, because cytokine measurements were restricted to serum, the observations should not be interpreted as fully reflecting local immune events occurring at the intestinal mucosa, where host–pathogen interactions primarily take place [27].

Comparison with other enteric pathogens further highlights the distinctive nature of this immune response. In neonatal broiler chickens infected with *Salmonella* Typhimurium, a broad and early systemic upregulation of cytokines and chemokines has been reported, including IL-6, IL-10, MIP-1β, MIP-3α, RANTES, M-CSF, and VEGF, reflecting a strong inflammatory response and active immune cell recruitment [26].

In contrast, the cytokine profile observed in the present study appears more moderate, delayed, and heterogeneous. These differences are likely attributable to pathogen-specific biological characteristics, since *Salmonella* is an invasive intracellular pathogen that triggers strong innate and cell-mediated immune responses, whereas *C. jejuni* behaves as a non-invasive coloniser in poultry, establishing persistent intestinal colonisation without inducing overt pathology [28]. Moreover, the different timing of sampling should also be considered, as the present study focused on later stages of infection corresponding to established colonisation. Therefore, comparisons between studies should be interpreted with caution, since differences in experimental design, sampling schedules, and immune parameters analysed may also have contributed to the observed variation in immune responses.

The present study has several limitations that should be considered when interpreting the findings. First, immune responses were evaluated exclusively at the systemic level using serum cytokines and antibodies, whereas local intestinal immune responses are likely to play a central role in the interaction between *C*. *jejuni* and the avian host. Consequently, the present data do not fully capture the immunological events occurring at the site of bacterial colonisation. Second, no functional assays were performed to further assess the biological implications of the observed cytokine modulation. In addition, correlations between bacterial loads and immune parameters were not investigated. Finally, host-related factors, including individual variability, genetic background, microbiota composition, and bacterial strain characteristics, may also have contributed to the heterogeneity of the immune responses observed. Future studies integrating systemic and mucosal immunological analyses, together with functional immune assays, would provide a more comprehensive understanding of the mechanisms underlying persistent *C*. *jejuni* colonisation. Accordingly, mechanistic interpretation based solely on the observed systemic immune responses should be interpreted carefully.

Overall, the findings describe an immune profile characterised by effective humoral activation, moderate systemic inflammatory and chemokine responses, and concurrent regulatory signalling, in the absence of strong Th1 polarisation. This pattern supports the concept of a stable host–pathogen equilibrium in broiler chickens, in which *C. jejuni* colonises the intestinal tract without causing overt pathology while still triggering measurable immune activation. The persistence of colonisation despite antibody production and cytokine modulation is consistent with a balanced host–pathogen interaction that favours persistence over bacterial clearance [27].

From a food safety perspective, this persistence of *C. jejuni* in broiler chickens despite measurable systemic immune activation contributes to sustained bacterial shedding and represents a major source of contamination during poultry processing. The longitudinal integration of bacterial colonisation with systemic humoral and cytokine responses provides insight into host–pathogen interactions during persistent infection and may support the development of targeted pre-harvest interventions, including vaccination strategies, microbiota-based approaches, and enhanced monitoring programmes aimed at reducing bacterial dissemination along the poultry production chain.

In conclusion, *C. jejuni* strain 8264ST-403 complex establishes persistent intestinal colonisation in broiler chickens while eliciting a measurable but limited systemic immune response. Although infection stimulated humoral immunity and cytokine modulation, these responses were not associated with effective bacterial elimination, supporting a stable host–pathogen equilibrium that favours long-term persistence in the avian host. The present findings indicate that systemic immune activation accompanies, but does not necessarily prevent, persistent intestinal colonisation in broiler chickens. A better understanding of the immune mechanisms associated with persistent *Campylobacter* colonization may contribute to the development of more effective control strategies aimed at reducing bacterial dissemination within poultry production systems and, ultimately, the risk of foodborne transmission.

## Data Availability

The original contributions presented in this study are included in the article. Further inquiries can be directed to the corresponding author.
